# Multisensor Network System for Wildfire Detection Using Infrared Image Processing

**DOI:** 10.1155/2013/402196

**Published:** 2013-06-16

**Authors:** I. Bosch, A. Serrano, L. Vergara

**Affiliations:** Signal Processing Group, Institute of Telecommunications and Multimedia Applications (iTEAM), Universitat Politècnica de València, Camino de Vera, S/N, 46022 Valencia, Spain

## Abstract

This paper presents the next step in the evolution of multi-sensor wireless network systems in the early automatic detection of forest fires. This network allows remote monitoring of each of the locations as well as communication between each of the sensors and with the control stations. The result is an increased coverage area, with quicker and safer responses. To determine the presence of a forest wildfire, the system employs decision fusion in thermal imaging, which can exploit various expected characteristics of a real fire, including short-term persistence and long-term increases over time. Results from testing in the laboratory and in a real environment are presented to authenticate and verify the accuracy of the operation of the proposed system. The system performance is gauged by the number of alarms and the time to the first alarm (corresponding to a real fire), for different probability of false alarm (PFA). The necessity of including decision fusion is thereby demonstrated.

## 1. Introduction

Conserving unique natural areas should be a priority for advanced societies in our time. One of the biggest threats faced by these natural areas is wildfire devastation. The unfortunate reality is that most of these areas are unprotected, or at most, only monitored during certain months of the year and then, only during certain times of day, leaving the nighttime periods more vulnerable without proper monitoring. The entire system suffers from teams of workers woefully ill equipped in terms of manpower and technology.

In response to these limitations, we have developed different ways to help these teams in their complex yet tedious task of forest monitoring. The literature has focused extensively on technical aspects of the problem with the aim of discovering solutions. 

Various authors have focused on solutions derived from specialized satellite infrastructure available today [1, 2]. Due to the nature of nongeosynchronous satellites [3], these proposals present four principal technical difficulties: the limited availability to cover the desired area, the effective resolution cell (taking into account the distances at which the sensors are positioned), (especially), the effective detection times and the times between satellite positioning.

Another option includes ground implementation, which entails designing specialized systems for the desired coverage area [4]. These designs employ different processing techniques that are typically divided into two major families (based on the type of information processed): the first is limited to collecting data with infrared sensors [5, 6]; the second encompasses working with visible images (such as [7, 8]), looking for specific types of fire in these images (as in [9] or [10]) and improving computer vision [11–13].

As part of ground implementation, additional consideration must often be given to expanding the inherently limited coverage area of these systems [14], thereby creating opportunities for wireless sensor networks as in [15, 16] with cameras or other specialized sensors [17, 18]. 

Another broad field includes the efforts of researchers to detect smoke [19] in visible images, [20, 21], to distinguish between the flame of the fire focus and smoke [22], and to use video to detect fires at night [23].

To address these issues, this paper presents the next step in the evolution of multisensor wireless network systems employed in terrestrial forest fire detection. This system has been under development for the last ten years as part of multiple research projects within the Signal Processing Group (GTS), part of the Institute of Telecommunication and Multimedia Applications (iTEAM) at the Universitat Politècnica de València (UPV). Our system exploits different expected characteristics of a real fire, including persistence and increases over time [24], in infrared images, while concurrently detecting smoke in visible images.

Research in the area of fire detection began with an initial processing scheme, as presented in [25]. It employed infrared radar as part of a linear scanning surveillance designed to detect wide-area, uncontrolled fires. The proposed scheme includes a linear predictor, and a subspace model with a prewhitening filter for the signal to be detected and introduces a simple procedure for improving linear prediction, as described in [26]. This scheme was applied to real infrared data collected by a passive infrared radar, located in a mountainous area in Southeast Spain (Alcoy, Alicante). Electronic range scanning and an azimuth mechanical system were likewise used. 

In [27], we presented a general scheme for the automatic detection of events in surveillance systems; it consisted of the initial basic scheme but extended to include nonlinear prediction and an increase detector. As part of the same project [27], exhaustive research was conducted on the design of the predictor, with the first theoretical considerations on the matched subspace detector and the increase detector being subsequently introduced. The need for decision fusion for the two detectors to make a final decision was likewise presented for the first time. Real-data experiments validated the interest of the proposed scheme. Results in a real operating system were shown, specifically those from several tests with real fires and from day-to-day operations in the Albufera Natural Park (Valencia).

Once the proposed schemes were installed in several real scenarios, we realized that the processing times of the various detectors needed to be considered. Consequently, detection algorithms were the focus of [28], with a special emphasis on the fusion of different decisions in order to exploit both the short-term persistence and the long-term increases found in uncontrolled fires. In [29, 30], we added a linear predictor to use a reference image for prediction, rather than previous images (used in earlier systems). System delays in alarm detection of controlled fire were also evaluated. Temporary evolution of false and true alarms is presented in [31, 32], part of a long-term performance evaluation carried out in the Font Roja Natural Park in Alcoy (Alicante, Spain).

In this paper, we focus on verifying the improvements made in the processing scheme for real fire signals.  Section 2 presents a description of the system, with real-data results presented in Section 3. Finally, conclusions about the improvements of the proposed scheme are then offered.

## 2. System Detector Scheme

The proposed system consists of a wireless sensor network with a central monitoring station. This sensor network is strategically positioned to significantly expand the effective coverage of the system, with several areas of overlap between the different coverages to verify alarms, especially when the distances increase considerably (tens of kilometers).

**Figure 1:**
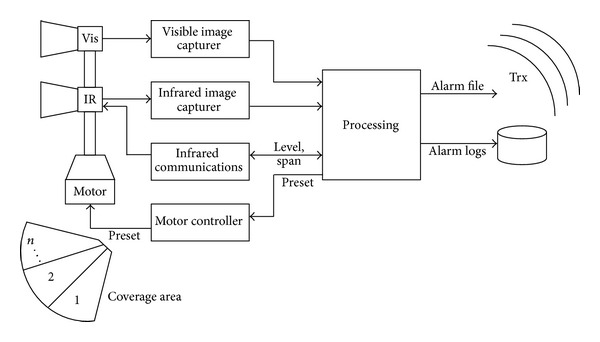
Sensor scheme.

Each sensor is comprised of two cameras (thermal and visible); a motor with different presets to sweep a larger area of coverage; and an integrated system of capture, processing and communication (see Figure 1). This sensor scheme allows autonomous monitoring of portions of the coverage area as well as in situ processing, generation, and transmission of alarms to the other elements in the wireless sensor network and to the central station. The said station can monitor the proper operation of the system and locate the position of each sensor with a geographic information system (GIS).

It is important to note that the system requirements are minimal: it is not necessary to use high-resolution cameras or show temperatures values, it does not require fast processing times, since it is better that the times between capture are seconds apart to have a margin for growth.

As mentioned above, the original system has been implemented and tested in real scenarios [27, 31] and shown to operate properly [32]. Thus, this paper proposes upgrading the original processing scheme with some improvements described in detail below and further verification with controlled fire experiments.

**Figure 2:**
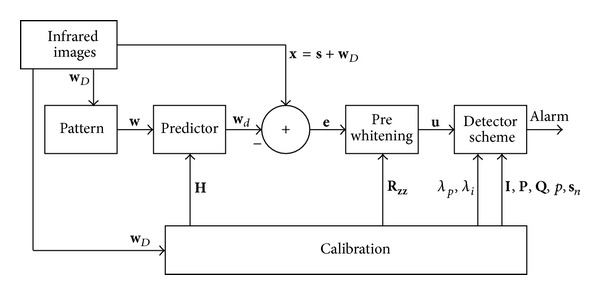
Processing Scheme.

The new processing scheme is shown in Figure 2, where each infrared image is converted into a matrix of pixels. Each pixel is associated with a resolution cell corresponding to certain coordinates of rank and azimuth; pixel-by-pixel processing is performed to then generate vectors describing the time history of each resolution cell.

The sensor motor is initially placed in one of the presets and, assuming no fire, the pattern **w** is calculated by acquiring a predefined number of images, which in turn, are used to generate the vector **w**_**D**_ (ideally composed only of noise). This vector **w**_**D**_ is sorted from low to high, the least representative of the extremes are removed, and the average of the remaining values is calculated, thereby yielding the searching pattern **w**. The system is also calibrated with the same images used in the pattern to generate the variables required in the subsequent processing stages. 

This pattern **w** is now introduced in the linear prediction stage, represented by matrix **H**, to obtain the estimated noise signal *x*_**p**_ = **H** · **w**.

Now, in normal operation, the infrared images are captured and generate the vector **x** = **s** + **w**_**d**_, composed of both signal **s** and noise **w**_**d**_. These values are then used to form vector **e** = **x** − **x**_**p**_, subtracting the previously estimated vector **x**_**p**_ from this vector **x**, which will ideally contain only signal **s** if they are predicted correctly.

This vector **e** has a Gaussian probability distribution [25], therefore a prewhitening stage must be performed using the matrix, **R** **z** **z**, to optimize the calculation of the threshold for a given PFA. Thus, we obtain the vector **u** = **R** **z** **z** · **e**, which is used as input for the subsequent detection stages.

We established four levels for risk of fire detection (ranging from low to high), with each corresponding to the following four alarms:

- Type 1: signal level alarm,
- Type 2: persistence in the signal level alarm (green in the figures of results),
- Type 3: increasing alarm (orange),
- Type 4: thermal saturation alarm (red).

The first type of alarm is designed to detect any change in the signal level. It is calculated from the vector **u** with a matched subspace filter (1), which uses an identity matrix **I** as a signal estimator. It thus becomes a simple signal-level detector to compare against a threshold, *λ*_*c*_, optimally calculated for a given PFA_*c*_ [25]:

(1) $$\begin{array}{c} c_{i}=u_{i}^{T}Iu_{i}≷\lambda_{c}. \end{array}$$

The second type of alarm is designed to observe the permanence of the change in the signal level, thus avoiding false alarms triggered by random changes or low persistence elements (e.g., a hot element moving into the infrared coverage area). It is calculated again from the vector **u** using a matched subspace filter, but now a projection matrix, **P**, is employed as a signal estimator, as designed in [25]. Assuming that the fire signature is inside a “low pass” subspace, the resulting estimator, *r* (2), with a *χ*_*p*_^2^ *distribution *(chi-square probability density function (pdf) with *p* degrees of freedom, where *p* is the subspace dimension), is compared with a second threshold, *λ*_*p*_, optimally calculated for a given PFA_*p*_ [25]:

(2) ri=uiTPui≷λp.

**Figure 3:**
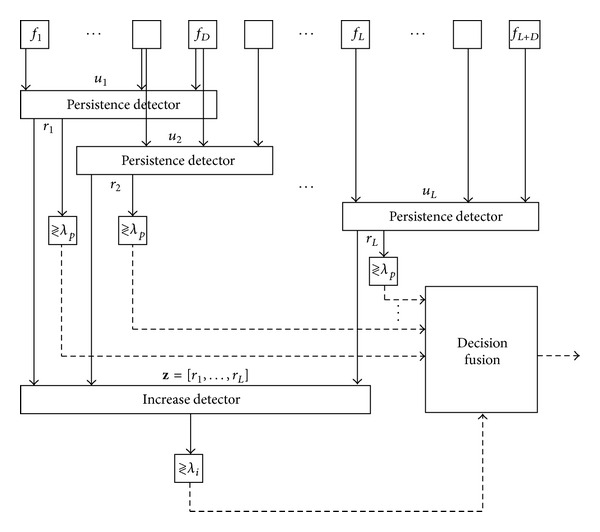
New detector scheme.

The third type of alarm is designed to detect the presence of increasing trends over a longer term. Thus, the observation period of time is increased from the *D* images used in the previous alarms to the *L* overlapped groups of *D* images, as seen in Figure 3. To accomplish this, we first generate an estimator vector *z* = [*r*_1_⋯*r*_*L*_]^*T*^, from the *L* previous *persistence detectors* results, **r**_**i**_, leaving only a margin of *nu* images without persistence detection to avoid sporadic decreases, according to the fusion rule implemented in [28]. 

Then, an increase estimator is generated from the decision fusion of the *L persistence detectors*, **z**, each compared with the threshold *λ*_*i*_. As in the previous cases, this threshold is optimally obtained from [27] for a required PFA_*i*_, according to the following expression (3):

(3) $$\begin{array}{c} \frac{z^{T}Q^{(n)T}{\left(Q^{\left(n\right)}Q^{(n)T}\right)}^{-1}s_{n}}{\sqrt{2ps_{n}^{T}{\left(Q^{\left(n\right)}Q^{(n)T}\right)}^{-1}s_{n}}}≷\lambda_{i}, \end{array}$$

where $s_{n}={\underset{L-n}{\underset{︸}{\left[1\cdots 1\right]}}}^{T}$, and the difference matrix is defined by **Q** ^(*n*)^ = **Q**_*L*−*n*+1_ ⋯ **Q**_*L*−1_ · **Q**_*L*_. Where matrix **Q**_*L*_ is defined by (4)

(4) $$\begin{array}{c} Q_{L}=\underset{L}{\underset{︸}{\left[\begin{array}{cccccc} -1 & 1 & 0 & \cdots & \cdots & 0\\ 0 & -1 & 1 & \cdots & \cdots & 0\\ & & \vdots & & & \\ 0 & 0 & 0 & \cdots & -1 & 1 \end{array}\right]\}}}L-1 \end{array}$$

Finally, the fourth type of alarm is the *thermal saturation alarm*, which is activated if the saturation level of the IR camera is surpassed.

These four types of detectors generate the corresponding four alarm types, but in practice it has been observed that they may be fused together (see detection scheme in Figure 3) as follows.

**Figure 4:**
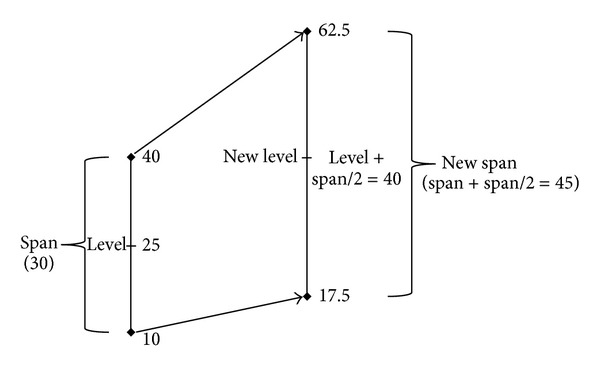
Change of level and span to leave sufficient margin of fire growth.

- Type 1, signal level alarm, is used as a requirement for all other alarms, thus preventing any observation of a low-level signal.
- Type 2, persistence level signal alarm, is used as a condition for checking the increase in the persistence in the type 3 alarm. With the *nu* parameter, it allows a number of controlled images without increased detection, and thereby avoids being too restrictive. A more comprehensive study on this condition can be found in [31].
- Type 3, increasing alarm, is activated when Type 1 (signal level alarm) and Type 2 (persistence alarm) have each been previously activated. This is a good indication of a possible source of fire.
- Type 4, thermal saturation alarm, is also used as an indication of fire if Types 1 and 2 have been previously activated. This is because, if the system is calibrated correctly, the level and span parameters of the IR camera must be artificially increased (e.g., 50% of the span, as shown in Figure 4). The fire level will then have a margin for growth. In Type 4, the thermal saturation level is only achievable if the signal level has been growing. In this case, Type 3 likewise fails because it does not possess any margin for growth.

## 3. Experiments

The system improvements were tested both in the laboratory, with known conditions, and in a controlled fire test. Additionally, results from a live-burn test in a real environment are shown to demonstrate the effectiveness of the proposed system.

In the laboratory experiments, the system was tested using a high power resistor supplied by a DC power source. Initially, with the power off, the system was calibrated and the pattern was generated. Subsequently, we activated the power supply and increased the voltage applied to the resistor; the increased radiated temperature simulated a possible source of heat.

In this case, we had to adjust the system parameters to simulate a fire at close range. Also, we had to add Gaussian noise onto the images with which the pattern **w** was calculated, because the span values were too small, and this caused numerical errors in the calculation of the calibration matrices. The added noise had a zero mean, and the variance was adjusted by taking into account the range of values of the camera signal (span). 

**Figure 5:**
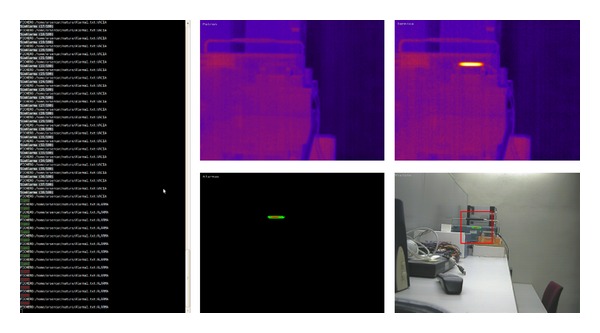
Capture of processing example in laboratory simulation.

Figure 5 shows captures of processing examples in the laboratory simulation. The left section displays a log with each iteration of the normal program operation. The right contains four images as follows: in the top left, the pattern used to detect the alarms is displayed; the top right shows the image captured by the thermal camera, as it is being processed; the bottom left shows the detected alarms; and the bottom right shows these detected alarms over the visible image. We can see how only the simulated fire is detected when generating the different types of alarms. Furthermore, on the bottom right, detected alarms are overlapped in the visible image, in order to locate them more easily. This process was performed using a projective transformation of the thermal image coordinates to the corresponding coordinates of the visible image. This transformation was calculated beforehand by manually defining equivalent locations in both images. In this case, alarms are not perfectly located on the visible image, because the distances with respect to the cameras are small, and the transformation error is large. Finally, a frame is also drawn to delimit the area in which all the alarms occur.

**Figure 6:**
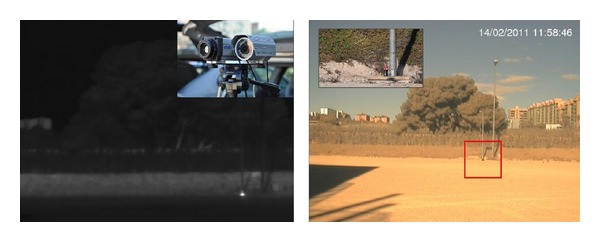
Controlled fire test in real environment.

A controlled fire test was then performed in a real environment. The simulated fire was generated in a small container at a distance of 100 m (Figure 6).

In Figure 6, the captured thermal image (FLIR ThermoVision A20-V, Focal Plane Array (FPA), uncooled microbolometer with spectral range: 7,5 to 13 *μ*m) can be seen on the left, and the visible image with overlapped alarms, on the right. Also, a closer view of the camera mounting and the source of the fire is shown in both images, respectively. The figure shows how the system properly detected the simulated fire and, in this case, correctly located it on the visible image. Several tests were performed with different levels of system sensitivity; in all cases, early detection of the fire was attained.

Finally, we tested the system in two real environments, with real controlled fires under firefighter supervision. In this case, the infrared data was recorded, and these recordings were subsequently processed in the laboratory to simulate a real-time operating environment, thereby allowing a comprehensive examination of the system performance to be conducted, based on different parameters.

**Figure 7:**
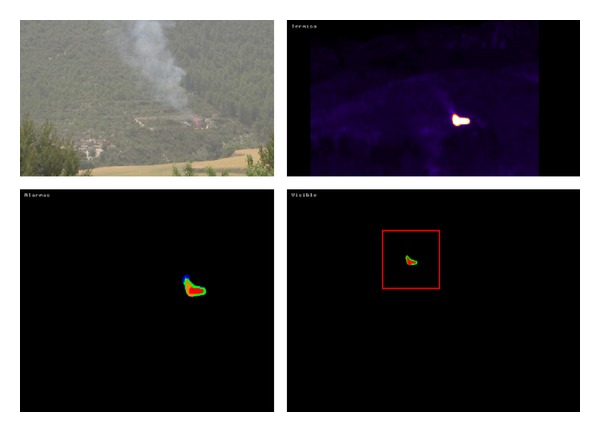
Example of controlled fire detection in real environment.

The first test was held in the Font Roja Natural Park in Alcoy (Alicante). It was a fire at a distance of 800 meters (Figure 7), and the fire can be seen in the image to start approximately 50 seconds into the recording. The second one was held in the Valencian town of Ayora. It was a fire at a greater distance (about 1500 meters), and the fire can be seen in the image to start approximately 30 seconds into the recording.

**Figure 8:**
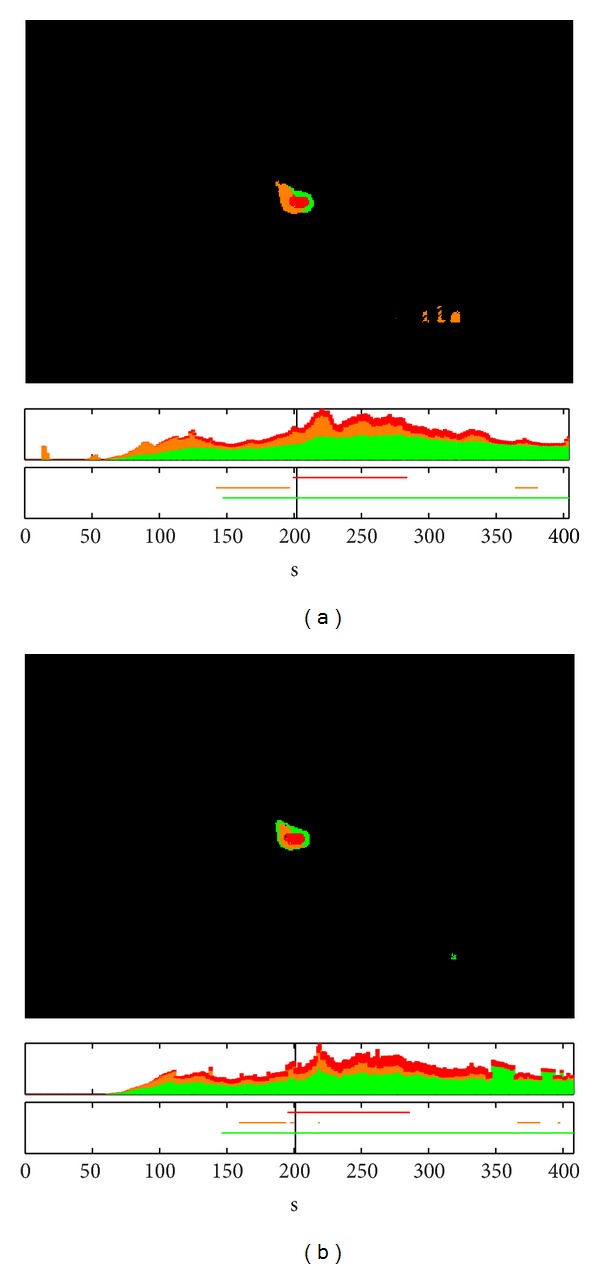
Fire alarm evolution for a fixed PFA with independent detectors (a) and fused detectors (b) in Font Roja.

**Figure 9:**
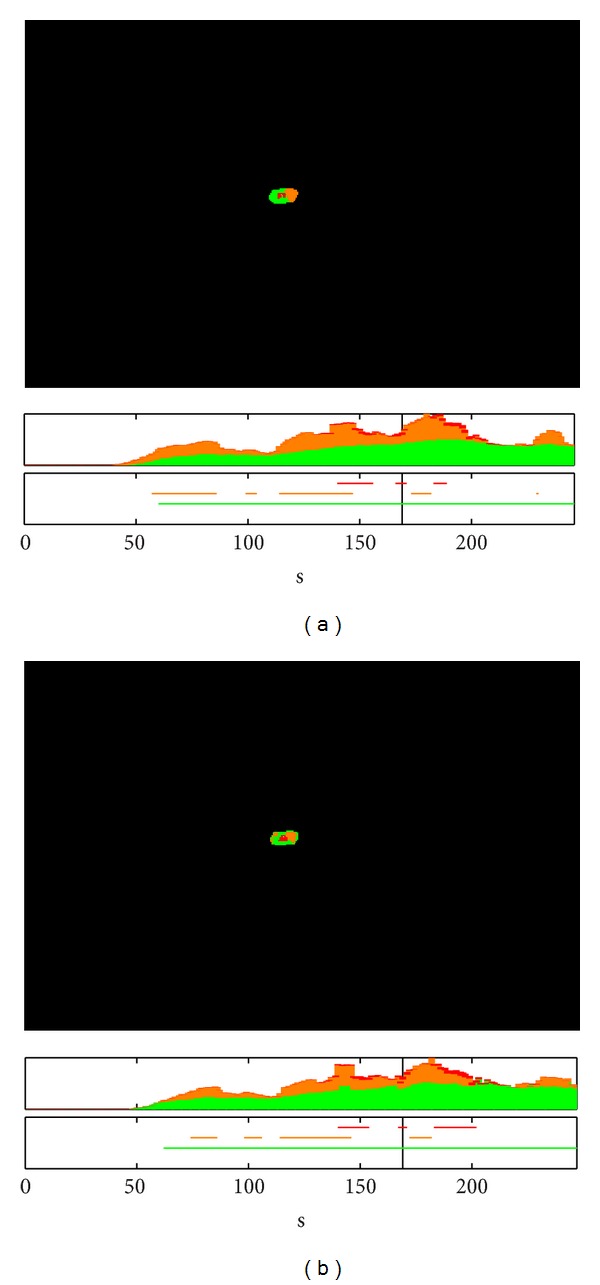
Fire alarms evolution for a fixed PFA with independent detectors (a) and fused detectors (b) in Ayora.

A comprehensive analysis of the alarm evolution was then carried out for a fixed PFA using independent detectors and the fusion rules implemented in the system (developed and discussed in Section 2). The results of this analysis of the experiments in Font Roja and Ayora are shown in Figures 8 and 9, respectively, as a comparison between independent detectors (a) and fused detectors (b). At the top of each subfigure, the state of the detected alarms for a given time is seen. The middle graph displays the evolution over time of the total number of alarms of different types for the whole image. The bottom subfigure graphs the evolution of alarms for a given pixel over time.

Looking specifically at the alarm images located at the top of these figures, the spatial distribution of the fire at a given instant (indicated by the vertical black line) can be deduced. From these, it can be verified that these are two fires moving spatially, because the *increasing alarms* are located at one end of the fire. Namely, in Figure 8, a fire is moving toward the left, while in Figure 9, displacement is to the right.

The bottom of each figure displays the time evolution for the different alarms of the same fire for a particular pixel, which has been chosen in order to observe all types of alarms. In both cases, it can be seen how, at this point, the fire temperature increased until reaching saturation. During this time, various types of alarms were generated, depending on whether the fusion of detectors was being used or not. 

In the nonfusion case, it can be observed how the *increasing alarms* may be activated before *the persistence alarm*, which could lead to an advantage in detection time. However, this may generate a greater number of false detections, as seen in the alarms that appear on the bottom-right portion of the alarms image in Figure 8(a) and, in the early detections appear in the evolution of the number of alarms in the same figure. These are not observed in the case of the fused scheme (see Figure 8(b)).

We can conclude that, for a fixed PFA, the probability of fire detection of the system can be greatly enhanced if these *increasing alarms* are preceded by *persistence alarms*, although a detection delay is introduced. That is, the necessity of using the implemented fusion rules and how this use introduces a significant improvement in a real environment are verified.

**Figure 10:**
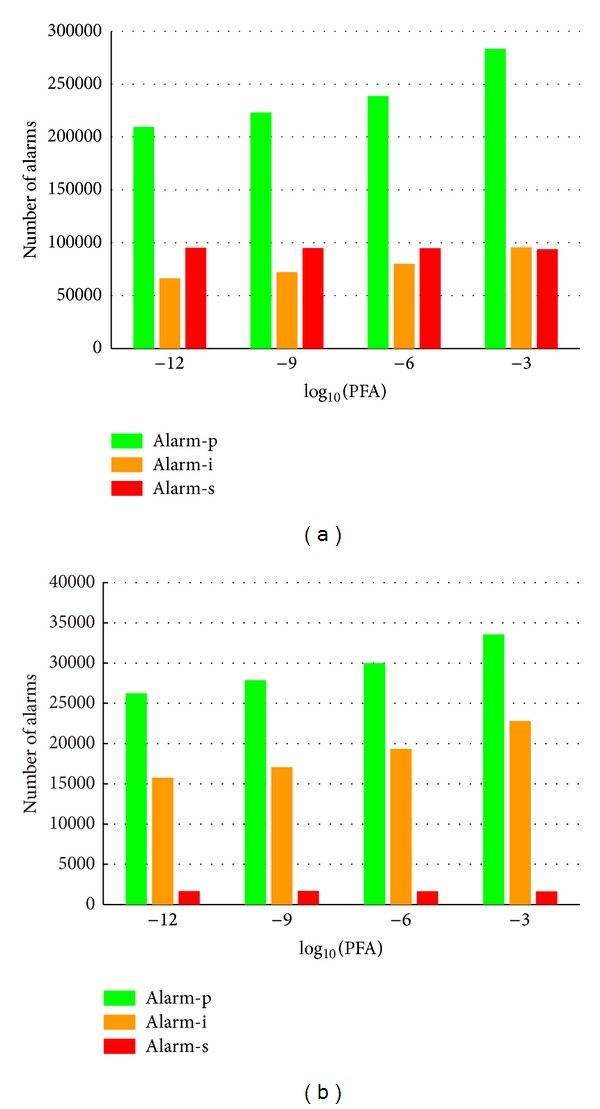
Number of alarms versus PFA, for different types of alarms, in Font Roja (a) and Ayora (b).

**Figure 11:**
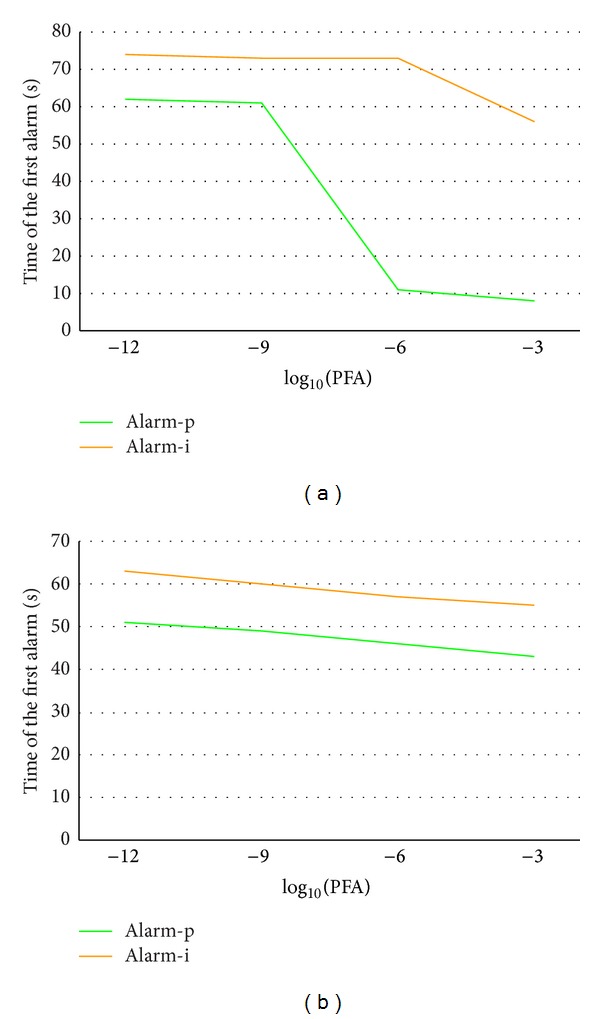
Time of the first alarm versus PFA for *persistence* (Alarm-p) and *increasing* (Alarm-i) alarms, in Font Roja (a) and Ayora (b).

While working with the fused detector scheme, an analysis was likewise performed on the evolution of the total number of alarms (Figure 10) and the time to the first alarm (Figure 11), depending on the required system PFA and the type of alarm.

The results shown in Figure 10 verify that, in practice, the number of *persistence* and *increasing alarms* rises as the PFA increases, independently of the *saturation* alarms. This is logical: as the PFA increases, more true or false alarms are present in the system. Thus, once again, the control exerted over the PFA is verified. We can also observe that the number of alarms is considerably greater in the case of Font Roja, since it was a fire at a smaller distance than in Ayora.

From Figure 11 we can verify in practice how, as the PFA increases, the time to the first alarm decreases. This is evident in the two graphs but particularly noticeable in the green line (*persistence alarms*) in Font Roja (Figure 11(a)), where the first alarms in the highest PFA were false, likewise observed in Figure 8(a). In Figure 11(b), this aspect is more clearly observed. The delay in the appearance of *increasing alarms* with respect to *persistence* ones is nearly constant and independent of the PFA.

## 4. Conclusions

We present the next step in the evolution of the multisensor wireless network system, based on infrared and advanced image sensors for automatic wildfire detection. This paper focuses on the description of the sensor and the processing scheme, highlighting the improvements in both. 

The different types of detectors are described and special emphasis is given to the decision fusion rules for the *persistence* and *increase detectors*, which can exploit short- and long-term characteristics expected in a real fire.

The functionality of the system is verified in diverse, controlled real-environment tests in order to authenticate the accuracy of the proposed system. Spatial and temporary evolutions of the alarms are likewise shown as part of an evaluation of the system in a real environment. Through a comprehensive analysis of different processing schemes, the necessity of including decision fusion is demonstrated. The performance of the system is also evaluated by measuring the number of alarms and the time to the first alarm corresponding to a real fire, for different PFA.

The results obtained reveal a high potential for this system in aiding human surveillance. Future research will include detecting smoke generated by a fire in the visible image.
